# Robotic airship for measurement and validation of a CERN detector stray magnetic field

**DOI:** 10.3389/frobt.2026.1926428

**Published:** 2026-10-16

**Authors:** Francesco Mazzei, Paolo F. Scaramuzzino, Luca Bernardi, Fabio Curti, Corrado Gargiulo

**Affiliations:** 1 Department of Physics, University of Rome Tor Vergata, Rome, Italy; 2 Experimental Physics Department, European Organization for Nuclear Research (CERN), Geneva, Switzerland; 3 Department of Systems and Industrial Engineering, University of Arizona, Tucson, AZ, United States

**Keywords:** ALICE detector, ArUco fiducials, blimp, CERN, Hall-effect sensor, robotic airship, stray magnetic field, harsh environments

## Abstract

The detector caverns of the Large Hadron Collider (LHC) at the European Organization for Nuclear Research (CERN) are harsh environments for robotic operation: confined, without satellite navigation, close to fragile hardware, and permeated by intense stray magnetic fields that can disturb onboard electronics, actuators, and sensors. Characterising these fields is a prerequisite for deploying robots in the caverns, and is also relevant to detector operation, since anomalous fringe fields can affect the surrounding infrastructure and particle tracking. Yet conventional approaches based on fixed sensors, mechanical systems, or manual surveys provide only sparse spatial coverage. This paper presents a robotic airship that combines a three-axis Hall-effect sensor with ArUco marker-based visual localisation to measure the three-dimensional stray field in the ALICE (A Large Ion Collider Experiment) detector cavern. Two campaigns were conducted with the magnets at nominal current: static measurements at twenty known positions, isolating sensor accuracy from localisation uncertainty, and dynamic flights over the same instrumented region. The measurements were compared with an electromagnetic simulation through a dedicated uncertainty-propagation framework. The simulation reproduces the transverse field components well (R^2^ = 0.86–0.98), whereas the vertical component shows a lower agreement (R^2^ = 0.75–0.79) and a systematic positive bias of 0.65–1.12 mT, more consistent with simplifications in the electromagnetic model than with measurement uncertainty. These results demonstrate the feasibility of aerial robotic platforms for stray field characterisation in large experimental facilities and lay the groundwork for a fully autonomous survey system.

## 1 Introduction

The next challenge at the European Organization for Nuclear Research (CERN) involves deploying robots inside the detector caverns at the four Large Hadron Collider (LHC) interaction points, which host the ATLAS, ALICE, CMS, and LHCb experiments (Aamodt et al., 2008; Aad et al., 2008; CMS Collaboration, 2008; LHCb Collaboration, 2015). Although robotic platforms have already been successfully deployed in the LHC tunnel environment for inspection and monitoring activities (Di Castro et al., 2018b; Di Castro et al., 2018a), these caverns present an additional critical constraint: powerful magnet systems generate intense stray fields that can interfere with electronics, actuators, and sensors onboard any robotic platform.

This challenge is expected to become substantially more severe in future facilities. The High-Luminosity LHC (HL-LHC) (Arduini et al., 2016) will employ stronger focusing magnets and further restrict direct human access. Looking further ahead, the Future Circular Collider (FCC-hh) (Benedikt et al., 2025) foresees no iron return yoke for the solenoid flux (Aleksa et al., 2022). Under this assumption, the stray field at the cavern wall ( r≈17.5 m) is expected to reach approximately 100 mT, falling below 5 mT only beyond 55 m from the detector (Benedikt et al., 2025). Such conditions, orders of magnitude more intense than in current LHC caverns, make magnetic compatibility a critical design driver for any future autonomous inspection platform.

Accurate characterisation of these stray fields is a prerequisite for any robotic platform operating in the cavern: without prior knowledge of the ambient field distribution, it is impossible to assess operational limits, model field-induced disturbances, or design field-aware navigation and control strategies. Beyond robotics, such a mapping campaign would also benefit experimental physicists: fringe fields extending beyond the detector volume can interfere with surrounding infrastructure and, if anomalous, compromise particle tracking and momentum reconstruction (Papaphilippou et al., 2003; Gohil et al., 2018). Yet no systematic effort has been dedicated to characterising or monitoring the stray field in the open cavern space, in contrast to the well-established mapping of the internal detector field (Fry et al., 2016; Hervé, 2000; Miele et al., 2001) (see Section 5). Real-time mapping could provide early warning of internal field anomalies, enabling rapid localisation of malfunctions before data quality is affected.

Traditional magnetic field mapping at CERN has relied on purpose-built mechanical systems with Hall-probe arrays (Aleksa et al., 2008; Klyukhin et al., 2008; Fry et al., 2016), which are effective within the detector bore but limited in spatial reach and accessibility in cluttered cavern environments. Fringe field characterisation has instead employed fixed sensors or manual surveys (Gohil et al., 2018), providing only sparse spatial coverage and requiring significant access coordination. Robotic platforms capable of autonomous navigation offer a compelling alternative to traditional fixed-sensor deployments, enabling continuous, high-resolution three-dimensional (3D) mapping without dedicated infrastructure or human access during operations (Almadhoun et al., 2016).

As part of the Experimental Physics Research & Development (EP-R&D) programme at CERN (Accettura et al., 2025; Gargiulo, 2025), aerial platforms have been identified as particularly suited for 3D cavern inspection and field mapping. Among them, blimps offer distinct advantages over multirotor drones: buoyant lift from helium enables extended flight endurance while inherently reducing collision risk, making them well-suited for safe indoor navigation near fragile detector components and for long-duration monitoring missions (Mazzei et al., 2023).

Beyond this internal research programme, airships have previously been used for magnetic mapping, but exclusively in outdoor aeromagnetic surveys of the quasi-uniform terrestrial field, from the pioneering Graf Zeppelin flight of 1931 (Raspopov et al., 2013) to more recent unmanned platforms (Kim et al., 2021). These efforts operated in weak, open-air, low-gradient conditions with GNSS available throughout, unlike the enclosed, GNSS-denied cavern considered here. UAV-based mapping studies, in turn, targeted comparatively weak indoor or industrial-scale fields using multirotor platforms (Lipovský et al., 2021; Kuevor et al., 2023), and did not validate against a high-fidelity electromagnetic model.

In the CERN detector caverns, however, the same magnetic environment that motivates aerial mapping simultaneously constitutes the primary hazard for the platform itself: the fringe field induces parasitic forces and moments on the electromagnetic actuators, degrading propulsion performance and destabilising flight. Addressing this dual role required a structured sequence of prior work: a feasibility study demonstrated stable blimp trajectory tracking in fringe fields representative of the FCC-hh cavern (Mazzei et al., 2023); an experimental campaign characterised the brushed coreless DC motors under external fields up to 0.4 T, identifying parasitic moments as the primary disturbance source (Mazzei et al., 2025); and a physics-based model of the propulsion–field interaction has been derived (Bernardi et al., 2026). The present work closes this loop by deploying the platform as a measurement instrument, delivering an *in situ*, spatially registered 3D reconstruction of a particle detector’s fringe field, validated quantitatively against the electromagnetic model across two complementary test campaigns. To the best of our knowledge, this work is the first to demonstrate airship-based mapping of a particle detector’s stray field.

Concretely, a full-scale electromagnetic model of the ALICE detector stray field cannot be fully trusted on simulation alone, as deviations from nominal geometry, material uncertainties, and nearby ferromagnetic structures cannot be captured without *in situ* measurements within the cavern volume. To this end, we present a robotic airship performing *in situ* 3D magnetic field measurements inside the ALICE detector cavern, combining a lightweight blimp platform, a three-axis Hall-effect sensor, and a vision-based localisation system using ArUco markers (Garrido-Jurado et al., 2014). Spatially registered measurements during two different test campaigns are compared quantitatively against a CST simulation of the ALICE magnet system, assessing the accuracy of the electromagnetic model and its suitability for disturbance-aware control, magnetic compatibility assessment, and magnetically aware motion planning.

The remainder of the paper is organised as follows. Section 2 describes the methodology adopted in this work. It is divided into four parts. Section 2.1 introduces the ALICE magnet system and the experimental cavern where the measurements were conducted. Section 2.2 presents the CST electromagnetic model developed to predict the magnetic field distribution. Section 2.3 describes the robotic airship, the magnetic sensing hardware, the data acquisition procedure, the localisation pipeline used to obtain spatially registered magnetic field measurements, and the two experimental campaigns carried out for comparison with the simulated field. Section 2.4 quantifies the uncertainty associated with the Hall sensor and the robot localisation, analyses its propagation to the measured magnetic field, and presents the metrics adopted to evaluate the agreement between the simulated and measured field distributions. Section 3 presents the CST simulation results, the outcomes of the two experimental campaigns, and their comparison with the simulated magnetic field. Finally, Section 4 discusses the main findings, the limitations of the proposed approach, and directions for future work.

## 2 Methods

This work addresses two objectives: (i) the demonstration of a robotic airship for *in situ* 3D magnetic field measurements in the ALICE cavern, extending the spatial coverage achievable with conventional static measurement campaigns; (ii) the experimental validation of a high-fidelity electromagnetic model of the ALICE magnet system using the collected measurements, establishing a magnetic environment model suitable for future disturbance-aware control and motion-planning studies.

### 2.1 The ALICE experiment and its magnetic infrastructure

ALICE (A Large Ion Collider Experiment) is a general-purpose heavy-ion detector at the CERN LHC, designed to study strongly interacting matter at extreme energy densities in nucleus–nucleus collisions (Aamodt et al., 2008). From the perspective of this work, the experiment is relevant primarily because of the powerful magnet systems that generate the stray magnetic field permeating the underground cavern in which the robotic airship operates.

The detector is located at Point 2 of the LHC in Saint-Genis-Pouilly, France. The UX25 underground cavern floor lies 52 m below the surface, with the beam line at 44 m depth. The cavern infrastructure includes a 20 t overhead crane, two levels of gangways running along its full length, and rack areas for electronics and services. Three distinct zones are identified along the beam axis: the A-side (low- β triplet and muon dipole compensator), the central region (L3 solenoid housing the central detectors), and the C-side (dipole magnet and forward muon system). These features (confined space, metallic structures, and strong magnetic fields) define the operating environment of the airship and motivate the design choices described in Section 2.3.

#### 2.1.1 L3 solenoid magnet

The dominant magnetic source in the cavern is the L3 solenoid shown in Figure 1, a room-temperature octagonal aluminium coil enclosed in a steel flux-return yoke. Originally commissioned in 1988 for the L3 experiment at the Large Electron–Positron (LEP) collider (Adeva et al., 1990) and subsequently adapted for ALICE, the solenoid generates a nominal axial field of 0.5 T over a volume of approximately 12 m in length and 11.8 m in diameter. Its principal characteristics are listed in Table 1. The fringe field extending beyond the yoke into the cavern volume constitutes the primary measurement target of this work.

**FIGURE 1:**
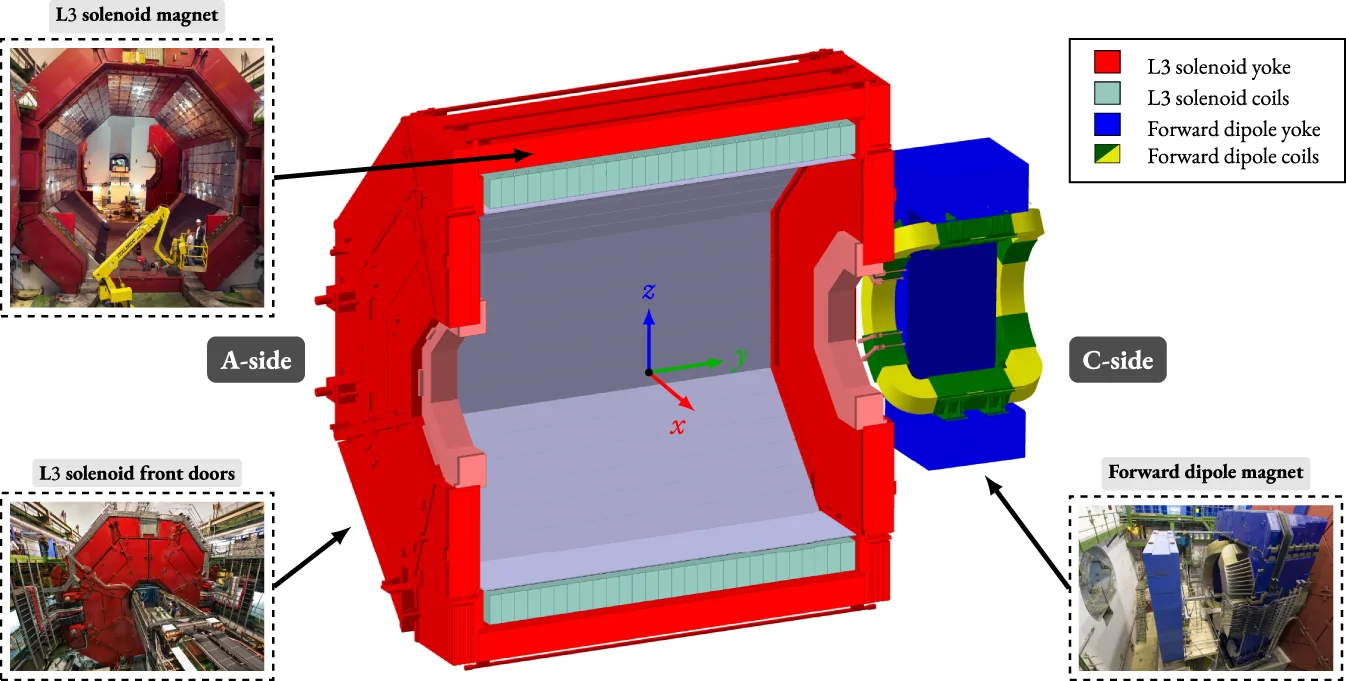
Simplified CAD model of the ALICE magnet system used in the CST simulation, showing the L3 solenoid and the forward dipole magnet. Insets show photographs of the actual detector components. The A-side and C-side labels indicate the two ends of the detector along the beam axis. Inset photographs: © CERN.

**TABLE 1:** Magnet characteristics of the L3 solenoid (Aamodt et al., 2008) and the forward dipole (Swoboda et al., 2006).

Parameter	Value	Unit
L3 solenoid		
Nominal flux density	0.50	T
Operating current	30.00	kA
Number of turns	168	–
Stored energy	150	MJ
Inner radius of coil	5930	mm
Overall dimensions (D × L)	15. 8×14.1	m
Total weight	7800	t
Forward dipole		
Operating current	6.00	kA
Stored energy	18.0	MJ
Nominal $B_{x}$ (centre of coils)	0.666	T
Field integral	3.04	Tm
Free pole gap	3.5	m

#### 2.1.2 Forward dipole magnet

A warm dipole magnet (Swoboda et al., 2006) is placed 7 m from the interaction point, adjacent to the L3 solenoid on the C-side of the cavern (Figure 1). It provides a nominal field of 0.67 T and a field integral of 3.04 Tm across a 3.5 m pole gap, serving the forward muon spectrometer. Its stray field contribution outside the magnet volume falls below 5 mT at the level of the cavern gangways. The coil characteristics are summarised in Table 1.

#### 2.1.3 Adopted reference frame

Throughout this work, spatial positions and field vectors are expressed in the ALICE coordinate frame $F_{\text{ALICE}}$ , as shown in Figure 1: the y -axis runs along the beam direction from the A-side toward the C-side, the z -axis points vertically upward, and the x -axis completes the right-handed triad, with the origin at the nominal interaction point. This frame is adopted consistently for the simulation output (Section 2.2), the localisation pipeline (Section 2.3.3), and the field measurements (Section 3).

### 2.2 Magnetic field simulation

An electromagnetic simulation of the ALICE magnet system was performed using CST Studio Suite 2024. Since the experimental measurements concern the field outside the detector volume, the model was set up to resolve the fringe field in the cavern region, while its overall correctness was verified against the nominal on-axis values (Section 3.1).

The model includes the relevant ferromagnetic components (the L3 return yoke and the forward dipole yoke), as well as the current-carrying coils. All other detector sub-systems (tracking, time-projection chamber, muon chamber, etc.) were treated as non-magnetic and therefore neglected. Component geometries were simplified from the official ALICE CAD model for simulation efficiency (Figure 1).

Both yokes were assigned the nonlinear, isotropic *B–H* curve of AISI 1050 annealed carbon steel, as retrieved from the Granta Selector material database. The coils were modelled as stranded conductors with a resistivity of $3\times 10^{-8}$ Ω m, consistent with aluminium. Excitation currents were set according to Table 1: 30 000 A through 168 turns for the L3 solenoid, and 6 000 A through two coils of 168 turns each for the forward dipole (3 sub-coils × 4 layers × 14 turns). The entire assembly was immersed in an air volume with open boundary conditions, which extend the domain virtually to infinity to correctly capture the stray field.

The mesh used tetrahedral elements with the following maximum element sizes: 450 mm for both the L3 return yoke and the forward dipole yoke, 350 mm for the coils, and 550 mm for the surrounding air volume (with automatic local refinement near component boundaries). The solver convergence threshold was set to a relative residual norm of $10^{-6}$ .

Starting from the numerical solution, a map of the magnetic flux density (BALICEsim) was generated by sampling the simulation domain on a uniform grid with a 200 mm step size, exporting spatial coordinates and vector components at each point. This map serves as the reference for comparison with the blimp measurements presented in Section 2.3.

### 2.3 Magnetic field measurement

#### 2.3.1 The robotic airship

The measurement platform is a custom helium-filled robotic airship designed for safe long-duration operation in proximity to the ALICE detector. All structural parts (envelope, gondola, and motor-fin supports) were fabricated from non-ferromagnetic materials (PLA and DEPRON foam) to prevent any magnetic attraction toward the L3 solenoid yoke. The envelope measures 1.70×0.65×0.65 m, and the complete platform has a total mass of ≈300 g. The main components of the platform are shown in Figure 2.

**FIGURE 2:**
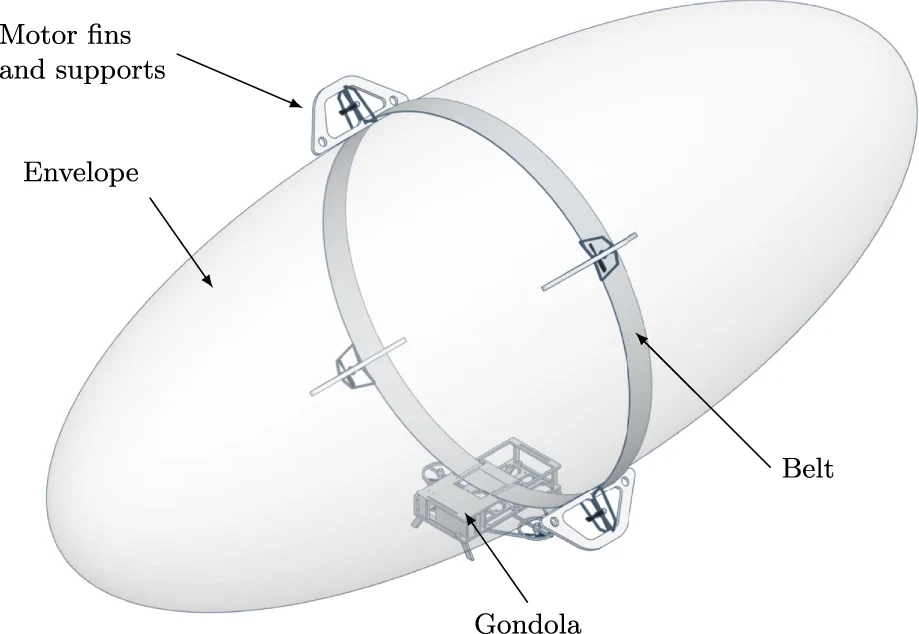
CAD rendering of the blimp prototype, highlighting the main structural components: envelope, gondola, belt, and propulsion fins.

The gondola, suspended below the envelope, houses the onboard electronics and the measurement payload. The primary instrument for this work is a TMAG5273 linear three-axis Hall-effect sensor (Texas Instruments/SparkFun), operated in the ±80 mT range and sampled at 10 Hz during flight. The sensor is read by an Arduino Portenta H7, which also acquires complementary onboard sensors including a 6 degrees of freedom (DoF) Inertial Measurement Unit (IMU), an environmental sensor (temperature, humidity, pressure), and a time-of-flight proximity sensor. Since each magnetic field measurement must be associated with a spatial position within the cavern, a Raspberry Pi Camera 3 Wide ( 1920×1080 px, 120 ° field of view) is also mounted in the gondola and operated by a dedicated Raspberry Pi Zero 2W, providing the image stream used by the ArUco-based localisation system described in Section 2.3.3.

Since the Hall-effect sensor and the camera are managed by two independent onboard computers, both data streams are timestamped independently and synchronised via Network Time Protocol (NTP) to a common time reference, limiting the temporal misalignment between field measurements and pose estimates during post-processing. All in-flight data are logged at 10 Hz in comma-separated values (CSV) format. The blimp is operated manually via a joystick ground station communicating over User Datagram Protocol (UDP) packets over the CERN Wi-Fi network; automatic fail-safe mechanisms (communication timeout, low battery) trigger immediate motor shutdown to prevent uncontrolled drift.

#### 2.3.2 Data acquisition

The SparkFun TMAG5273 is a low-power, high-precision 3D Hall-effect sensor from Texas Instruments, communicating via Inter Integrated Circuit (I^2^C). It offers a configurable linear magnetic range of ±40 mT or ±80 mT, with an integrated 12-bit Analog-to-Digital Converter (ADC) and on-chip COordinate Rotation DIgital Computer (CORDIC) angle calculation with gain and offset correction. The sensor was operated in its default configuration: ±80 mT range and no internal averaging (CONV_AVG=000b, corresponding to single-sample conversion at ∼ 10kSPS for three axes).

During post-processing, magnetic field samples BmeasS(tarduino) are associated with pose estimates via a nearest-neighbour temporal matching scheme: for each ArUco pose timestamp $t_{\text{rpi},k}$ , the Arduino sample, whose timestamp minimises $\left|t_{\text{arduino}}-t_{\text{rpi},k}\right|$ , is selected. Pairs whose minimum temporal separation exceeds 200 ms are discarded to avoid associating measurements acquired at substantially different instants.

#### 2.3.3 Sensor localisation

To associate each magnetic field measurement with a spatial position within the cavern, the pose of the Hall-effect sensor must be estimated in real time and expressed in the ALICE reference frame. This is achieved through a vision-based localisation system relying on ArUco fiducial markers (Garrido-Jurado et al., 2014), i.e., square binary patterns designed for robust visual detection and pose estimation, distributed across the cavern surfaces and imaged by the onboard camera.

Throughout this section, we adopt the homogeneous matrix convention, in which a transformation matrix ^*A*^ $T_{B}$ compactly encodes both the position and orientation of frame $F_{B}$ with respect to frame $F_{A}$ .

The flight area geometry was extracted from the official ALICE CAD model, from which all suitable surfaces for marker placement were identified. A total of 82 markers from the aruco.DICT_4X4_100 dictionary were generated using OpenCV, printed on continuous paper rolls, and arranged in strips to cover the selected surfaces (see Figure 3). A JSON vocabulary file associates each marker ID with the 3D coordinates of its centre in the global reference frame $F_{G}$ , with marker 0 chosen as the origin.

**FIGURE 3:**
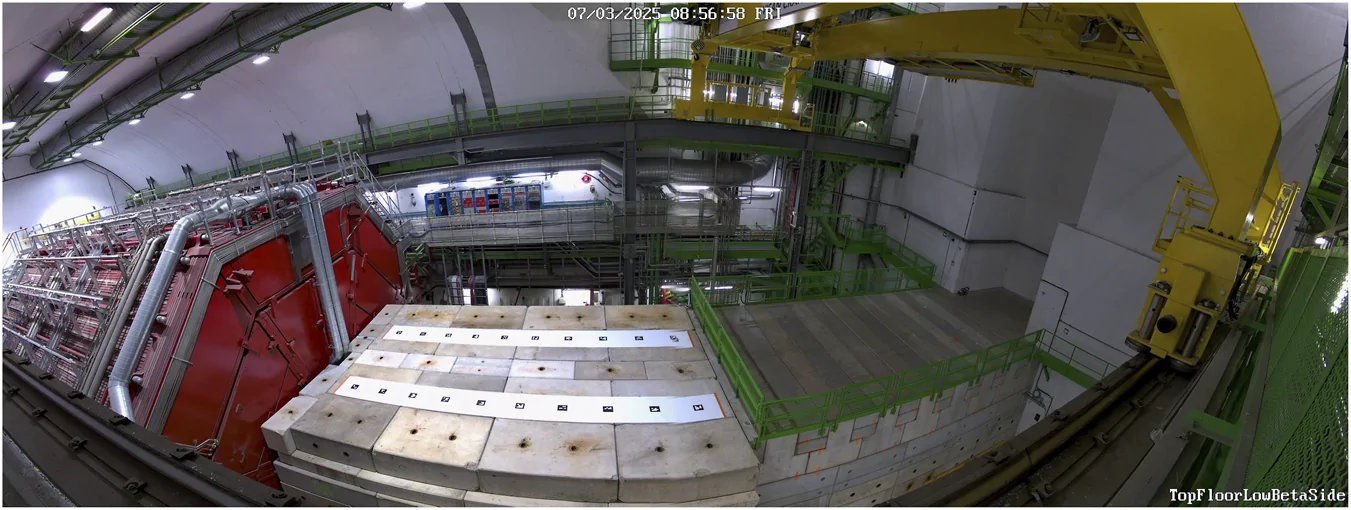
Picture of ArUco markers placed on the concrete blocks in front of the ALICE L3 solenoid doors.

Since all markers were printed and mounted with the same in-plane orientation relative to $F_{G}$ (i.e., the local marker axes are parallel to those of the global frame across all strips), the rotation matrix ^*G*^ $R_{\text{Ar},i}$ is the identity for every marker i . Consequently, the transformation ^*G*^ $T_{\text{Ar},i}$ stored in the JSON vocabulary reduces to a pure translation,

TAr,iG=I3tAr,iG0⊤1, (1)

where tAr,iG is the position of the centre of marker i relative to marker 0, the origin of $F_{G}$ , as recorded in the JSON file.

The marker strips were deployed along the joint lines between the concrete shielding blocks in front of the L3 solenoid, whose installation is governed by an official procedure constraining their position with respect to the detector alignment. Within a strip the marker spacing is fixed by the printed layout, while the placement of the strip as a whole was performed manually against the joint line and is estimated to be accurate to within a few centimetres. The translation component ^ALICE^ $t_{G}$ , i.e., the position of the origin of $F_{G}$ with respect to the ALICE interaction point, could not be measured directly, as the interaction point lies inside the detector volume, and was therefore derived from the as-built CAD model; the markers were then positioned in the cavern according to this CAD-based layout, and their alignment was verified on site. Accordingly, ^ALICE^ $T_{G}$ is treated as a fixed, ideal transformation, since no documented tolerance is available, and is not propagated in Section 2.4.2.

The transformation from the global ArUco frame, used by the localisation system, to the ALICE global frame $F_{\text{ALICE}}$ , adopted in the CST electromagnetic simulation, is a pure translation:

TGALICE=I3tALICEG0⊤1,tGALICE=−6.53−29.0−1.70 m. (2)

Camera calibration was performed prior to deployment using a standard checkerboard procedure, yielding a mean reprojection error of 0.033 px. Given an image acquired by the onboard camera, the marker detection stage identifies all visible markers using OpenCV’s ArucoDetector. For each detected marker, the algorithm returns the pixel coordinates of its four corners and its unique ID, the latter used to retrieve the corresponding 3D position from the JSON map. Detections were accepted only when the reprojection error remained below 1 px and the marker normal vector was oriented toward the camera, discarding geometrically ambiguous solutions. When multiple markers were simultaneously visible, the estimate with the lowest reprojection error was retained, improving robustness against partial occlusions.

Pose estimation reduces to the Perspective- n -Point (PnP) problem (Hartley and Zisserman, 2003): for each detected marker i , solvePnP matches the observed corner pixel coordinates against the known physical corner positions ±s/2 , where s=0.20 m is the marker side length, and returns the transformation ^cam^ $T_{\text{Ar},i}$ , which maps the local ArUco frame into the camera frame. This is inverted to obtain TAr,icam . Since each marker is defined within the global frame $F_{G}$ , the camera pose in the global frame is recovered by chaining transformations:

TcamG=TAr,iG TcamAr,i, (3)

where TAr,iG is the pure translation of Equation 1, encoding the known 3D position of marker i in $F_{G}$ , as stored in the JSON vocabulary file. The camera pose in the ALICE frame then follows by applying the constant transformation derived from the CAD model, as given in Equation 2:

TALICEcam=TALICEG TcamG. (4)

The pose of the Hall-effect sensor in $F_{\text{ALICE}}$ is then obtained by applying the fixed rigid transformation ^cam^ $T_{S}$ between the sensor frame $F_{S}$ and the camera frame $F_{\text{cam}}$ , determined from the gondola CAD model:

TALICES=TALICEcam TcamS, (5)

where

RcamS=01010000−1,tcamS=0.030.160 m. (6)

Substituting Equations 3, 4 into Equation 5, the full transformation chain yielding the position and orientation of the sensor frame in $F_{\text{ALICE}}$ is:

TALICES=TALICEG TAr,iG TAr,icam TcamS. (7)

Finally, the magnetic field vector BSmeas , measured in the sensor frame, is rotated into $F_{\text{ALICE}}$ for comparison with the CST simulation:

BALICEmeasx,y,z=RALICES BSmeasx,y,z, (8)

where RALICES is the rotation submatrix extracted from ^ALICE^ $T_{S}$ at each detection timestamp.

#### 2.3.4 Experimental campaigns

The CST electromagnetic model of the ALICE magnets, described in Section 2.2, is a necessary simplification of the real detector environment: it does not capture local ferromagnetic structures, construction tolerances, or the residual influence of neighbouring magnets not included in the simulation geometry. To quantify the resulting discrepancy between simulation and reality, two complementary experimental campaigns were conducted.

The first campaign consisted of static measurements at known locations, in which the Hall sensor was positioned at predefined reference points inside the cavern. By eliminating the localisation uncertainty associated with the ArUco system, these measurements were used to isolate the contribution of the Hall sensor and to estimate the discrepancy between the measurements and the CST model.

A rigid platform was designed and 3D-printed with the same footprint as an ArUco marker (0.2 m×0.2 m) . The same Hall-effect sensor (TMAG5273) used on board the blimp was mounted at the geometric centre of the platform, so that the sensor reference frame coincides with the ArUco coordinate system and the sensor position is directly traceable to the global frame $F_{G}$ . An operator placed the assembly on a selected ArUco marker; a second operator triggered a 30 s static acquisition. Twenty markers within the instrumented region were measured in this way, yielding, for each marker, a 30 s time series of $\left(B_{x},B_{y},B_{z}\right)$ from which the mean field value was computed.

Measured positions were transformed into the ALICE reference frame via Equation 2. The CST field values at each measurement point were obtained by linear interpolation of the simulation grid using three scatteredInterpolant objects built for $B_{x}$ , $B_{y}$ , and $B_{z}$ respectively.

The 20 measurement points form a 2×10 grid in two parallel strips along the $y_{\text{ALICE}}$ axis, at lateral positions $x_{G}\approx 5.19$ m (even IDs 24–42) and $x_{G}\approx 8.27$ m (odd IDs 25–43), all at a common height $z_{G}=4.45$ m, with consecutive markers spaced approximately 0.914 m apart along $y_{\text{ALICE}}$ (see Figure 3).

The second campaign consisted of dynamic flight tests, in which the blimp acquired magnetic field measurements while navigating through the cavern (see Figure 4). In this case, the comparison with the simulated field is affected by both the Hall-sensor uncertainty and the ArUco-based localisation uncertainty.

**FIGURE 4:**
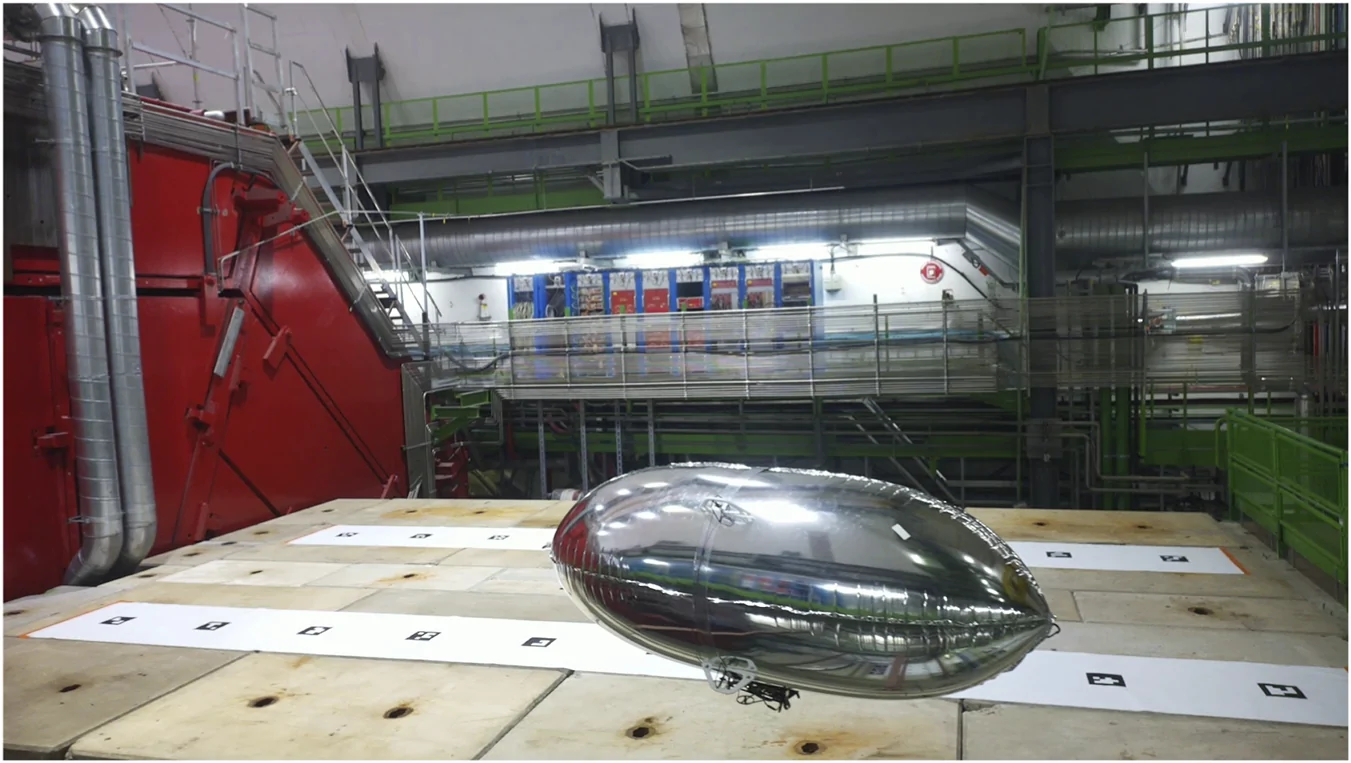
Picture of the blimp flying in the ALICE cavern during the dynamic flight tests.

The primary flight area was located above the concrete shielding blocks adjacent to the detector access doors (see Figure 3), providing sufficient clearance for safe blimp operation while remaining within the region of interest for magnetic field mapping. All flights were performed manually by an operator using a joystick-based ground control station. The blimp was flown along the same two marker strips used in the static campaign, maximising the fraction of frames with a valid pose estimate.

Four flight sessions were conducted on two separate days (4 February and 22 May 2026), with the ALICE magnet system powered at nominal current and both the L3 solenoid and the forward dipole operating at negative polarity.

The blimp explored a volume covering approximately $x_{\text{ALICE}}\in \left[-3.7,\;2.6\right]$ m, $y_{\text{ALICE}}\in \left[-18.5,\;-7.6\right]$ m, and $z_{\text{ALICE}}\in \left[3.7,\;6.6\right]$ m, corresponding to the region in front of the concrete shielding blocks on the A-side of the detector.

### 2.4 Validation procedure

Section 2.2 describes the generation of the simulated magnetic field map, denoted by ^ALICE^ $B_{\text{sim}}$ , while Section 2.3 presents the robotic measurement system and the processing pipeline used to obtain the measured magnetic field vectors, denoted by ^ALICE^ $B_{\text{meas}}$ . To validate the simulation, the two datasets are compared at the spatial coordinates estimated by the localisation system. Since the CST field map is available only at discrete grid locations, a scattered interpolant is used to evaluate the simulated field ^ALICE^ $B_{\text{sim}}\left(x^{*},y^{*},z^{*}\right)$ at arbitrary query points $\left(x^{*},y^{*},z^{*}\right)$ corresponding to the measurement locations. The difference between measured and simulated magnetic field vectors is then defined as:

ΔB=BALICEmeas−BsimALICE. (9)

Figure 5 summarises the processing pipeline used to obtain and compare measured and simulated magnetic field vectors in the ALICE reference frame $F_{\text{ALICE}}$ . The uncertainty sources associated with each processing stage are highlighted in red, including contributions from ArUco-based localisation, Hall-sensor acquisition, coordinate transformations, and the CST electromagnetic model.

**FIGURE 5:**
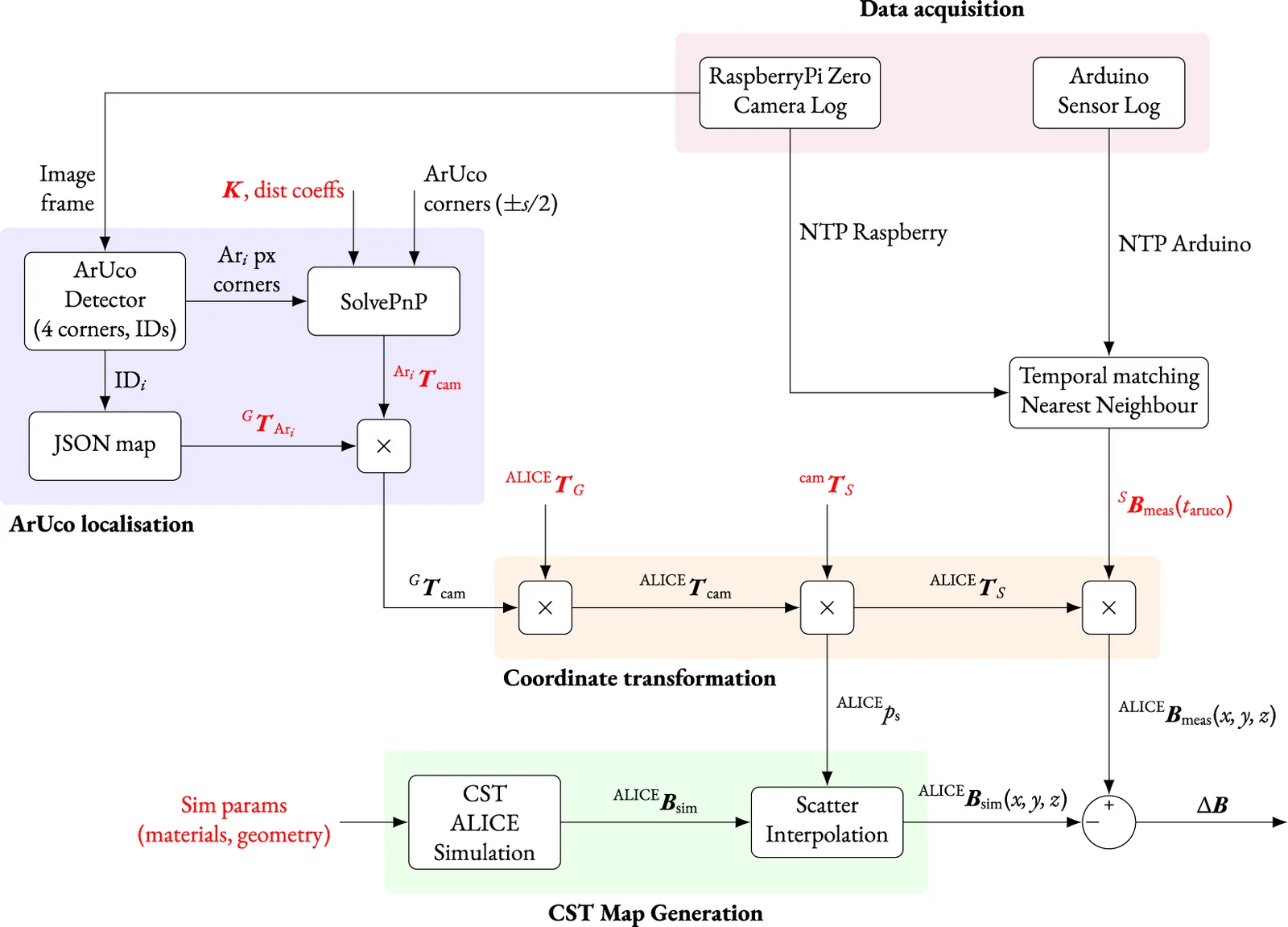
Processing pipeline used to validate the CST electromagnetic model against robotic field measurements. Sensor measurements are transformed into the ALICE reference frame $F_{\text{ALICE}}$ and matched to the corresponding simulation values through spatial interpolation of the CST field map. The resulting residual vector ΔB quantifies the agreement between measurements and simulation.

Specifically, the ArUco localisation stage is affected by uncertainties in the camera intrinsic calibration (camera matrix K and distortion coefficients), residual errors in the solvePnP pose estimation (TAricam) , and inaccuracies in the placement of the marker strips within the global reference frame (TAriG) . The spacing between markers within each strip is fixed by the printed layout, whereas the relative placement of the strips was established manually during installation and therefore contributes to the uncertainty on ^G^ $T_{{\text{Ar}}_{i}}$ . For the purpose of uncertainty propagation, the combined effect of these contributions is modelled as an uncertainty on the estimated camera pose ^G^ $T_{\text{cam}}$ , which can be directly characterised experimentally.

The transformation chain introduces further uncertainty through the transformation between the global ArUco reference frame $F_{G}$ and the ALICE reference frame $F_{\text{ALICE}}$ (TALICEG) , which is derived from the CAD model, with the marker placement verified on site inside the cavern. This contribution is not propagated: the transformation is fixed for the entire campaign and no documented tolerance is available for it, so no standard uncertainty can be assigned. The discrepancy between the CAD geometry and the as-built infrastructure is expected to remain within the construction tolerances of the ALICE detector and cavern, and would manifest as a bias common to all measurement points rather than as a point-to-point dispersion.

Additional errors arise from the camera-to-sensor transformation (TcamS , Equations 5, 6), whose nominal transformation is obtained from the gondola CAD model but is affected by mechanical assembly tolerances. Owing to the millimetre-level precision of the mechanical assembly, its effect is expected to be small relative to the other contributions; being a fixed transformation without a documented tolerance, it is likewise treated as ideal and omitted from the propagation analysis.

Temporal misalignment between Raspberry Pi pose estimates and Arduino Hall-sensor measurements is minimised through NTP synchronisation. Given the low cruising speed of the blimp, the resulting spatial error is expected to be negligible compared with the other uncertainty sources and is likewise excluded from the uncertainty quantification.

Finally, the CST model is affected by uncertainties associated with material properties, geometric simplifications, and assumptions introduced during the construction of the electromagnetic model. These modelling uncertainties are not propagated explicitly in the present analysis, as the primary objective is to quantify the discrepancy between the simulation and the experimental measurements. The observed residuals will be subsequently used to refine the electromagnetic model and reduce these uncertainties.

In summary, among all the contributions, the Hall-effect sensor and the pose accuracy of the ArUco-based localisation system are identified as dominant and detailed in Section 2.4.1.

#### 2.4.1 Uncertainty quantification

In order to quantify the standard deviations associated with the variables retained in the uncertainty model and to propagate their effect to the measured magnetic field vector ^ALICE^ $B_{\text{meas}}$ , the covariance matrices associated with the pose estimation, $\Sigma_{\text{pose}}$ , and with the Hall sensor measurements, $\Sigma_{\text{hall}}$ , are first derived. These matrices are subsequently combined and propagated to obtain the total measurement covariance matrix, $\Sigma_{\text{tot}}$ , which represents the overall uncertainty of the magnetic field measurements. The diagonal elements of $\Sigma_{\text{tot}}$ provide the variances of the measured field components $B_{x}$ , $B_{y}$ , and $B_{z}$ , from which the corresponding standard deviations are obtained. These standard deviations are then used to construct the ±1σ uncertainty bands around each measured field component. Finally, the magnetic field values predicted by the CST simulation at the same measurement locations are compared against these uncertainty bands. Agreement within the bands indicates consistency between the simulation and the experimental measurements, whereas systematic deviations outside the bands suggest that the simulation model may require further refinement or parameter updating.

##### 2.4.1.1 Hall sensor accuracy

The total measurement uncertainty on a single field reading $B_{\text{meas}}$ is dominated by three independent contributions (Texas Instruments, 2026). Following the GUM Type B evaluation procedure (JCGM, 2008, §4.3.7), each manufacturer-specified error bound without stated distributional information is treated as the half-width of a rectangular (uniform) distribution, and converted to an equivalent standard uncertainty by dividing by $\sqrt{3}$ .

The first is a sensitivity error $\varepsilon_{s}$ , a multiplicative gain uncertainty with a guaranteed maximum bound of ±20% (SENSER, maximum specification) arising from the LSB-to-field conversion factor. Under the rectangular-distribution assumption, the corresponding standard uncertainty is

$$\sigma_{\text{sens},i}=\frac{0.20}{\sqrt{3}}\times |B_{\text{meas},i}|\approx 0.1155\times |B_{\text{meas},i}|.$$ (10)

The second is a zero-field offset $B_{\text{off}}$ with a guaranteed maximum bound of ±1000 μ T, which under the same assumption contributes a fixed additive standard uncertainty

$$\sigma_{\text{off}}=\frac{1000\;\mu T}{\sqrt{3}}\approx 577.4\;\mu T,$$ (11)

independent of the applied field. The third is intrinsic RMS noise, already reported by the manufacturer as a 1σ standard deviation (CONV_AVG=000b, no averaging): 125 μ T for the x and y axes and 68 μ T for the z -axis. This term requires no further conversion.

This combination follows the error-budget methodology recommended by the manufacturer itself (Texas Instruments, 2026, Section 7.1.5, Equation 19), which likewise combines sensitivity error, offset, and RMS noise in quadrature.

Combining these three contributions in quadrature yields the total per-measurement uncertainty:

$$\sigma_{\text{hall},i}=\sqrt{\sigma_{\text{sens},i}^{2}+\sigma_{\text{off}}^{2}+\sigma_{\text{noise},i}^{2}},\;i=x,y,z,$$ (12)

from which the covariance matrix is constructed as

$$\Sigma_{\text{hall}}=diag\left(\sigma_{\text{hall},x}^{2},\;\sigma_{\text{hall},y}^{2},\;\sigma_{\text{hall},z}^{2}\right).$$ (13)

For the static test, $B_{\text{meas},i}$ in Equation 12 is obtained as the mean magnetic field value ${\bar{B}}_{\text{meas},i}^{\;k}$ , computed by averaging all raw samples acquired within the corresponding 30 s measurement window at each of the k known positions, whereas for the dynamic test it is computed directly from the instantaneous measured value.

##### 2.4.1.2 Localisation accuracy

To quantify the positional uncertainty introduced by the vision-based pose estimator at each field measurement timestamp, a dedicated validation campaign was conducted in a controlled indoor environment equipped with a Qualisys motion-capture (MoCap) system, as shown in Figure 6. ArUco-based pose estimates were compared against MoCap ground truth over multiple free-flight sessions.

**FIGURE 6:**
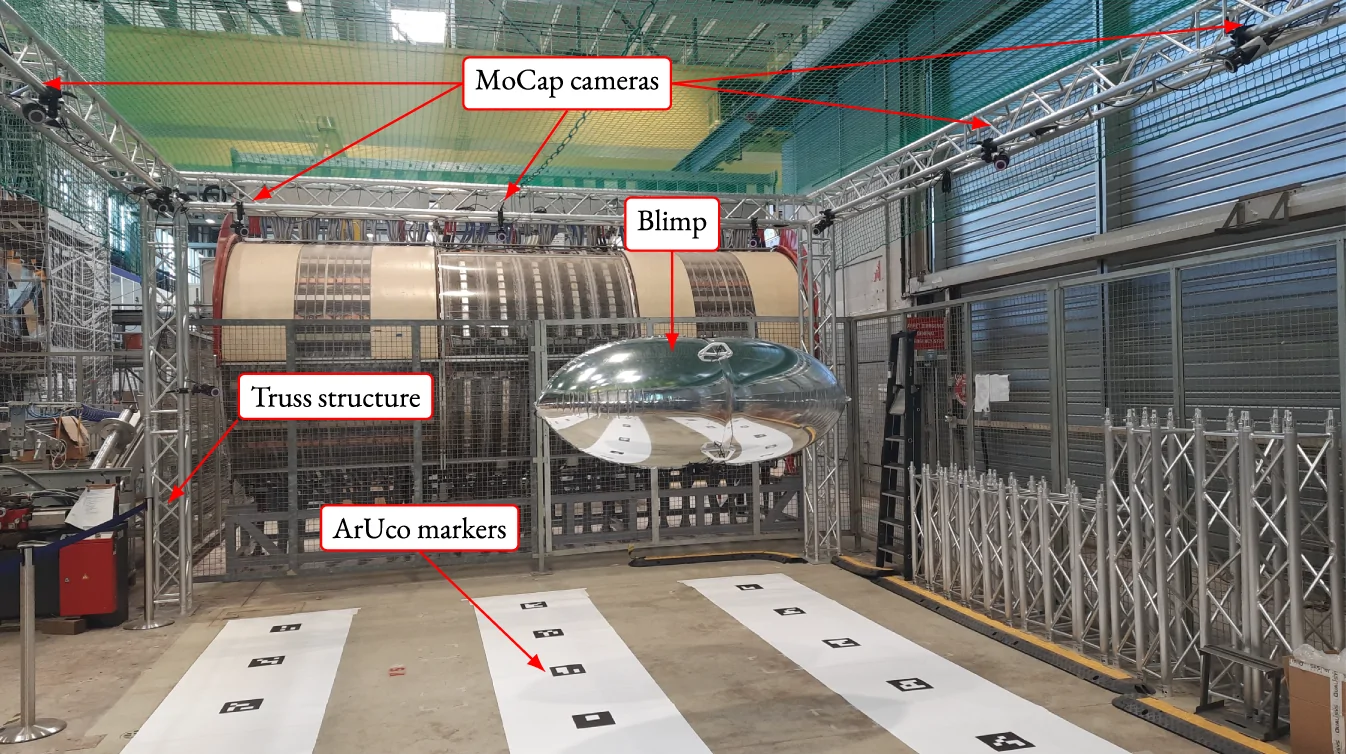
Picture of the blimp inside the motion-capture validation area. ArUco marker strips are placed on the floor; cameras are mounted on the truss structure.

The marker layout was designed to replicate, as closely as possible, the deployment conditions used in the ALICE cavern. A total of 21 ArUco markers (DICT_4X4_100, side length s=0.20 m) were arranged on the floor in three parallel strips of seven markers, with an inter-marker spacing of approximately 0.914 m along each strip, covering an area of roughly 3.86×5.59 ${\text{m}}^{2}$ . The blimp was flown freely across N=14 sessions at varying altitudes and lateral positions within the instrumented area, accumulating n=1398 valid detection frames.

A Mahalanobis-distance outlier rejection was then applied separately to the position error vector $e^{\text{pos}}={\left[e_{x},e_{y},e_{z}\right]}^{⊤}$ and the attitude error vector $e^{\text{att}}={\left[e_{\phi },e_{\theta },e_{\psi }\right]}^{⊤}$ , under the assumption that each is approximately multivariate Gaussian; a frame was retained only if both squared Mahalanobis distances fell below the $\chi_{3,0.95}^{2}=7.815$ threshold. This removed 80 position outliers (5.72%) and 67 attitude outliers (4.79%) , for a combined 113 frames (8.08%) discarded and a cleaned pool of n=1285 frames (91.9%) used for all subsequent analysis. The observed rejection rates are consistent with the $\chi_{3,0.95}^{2}$ criterion applied to a nominally Gaussian population ( ≈5% per error vector and ≈9.8% jointly), indicating that the procedure removes tail contamination without truncating a substantial fraction of legitimate measurements.

One-sample Kolmogorov–Smirnov tests on the standardised residuals confirmed that the heavy-tailed contamination present before cleaning (normality rejected for five of the six components, $p\leq 1.9\times 10^{-2}$ , the only exception being $e_{\theta }$ at p=0.20 ) was removed for all components except the rotation about the camera z axis (normality no longer rejected for the five retained, p≥0.058 , against $p=4.8\times 10^{-3}$ for $e_{\psi }$ ). To assess the practical magnitude of the residual departure, the sample skewness and kurtosis of the cleaned $e_{\psi }$ residuals were computed ( γ=−0.13 , κ=2.26 ), indicating a mildly platykurtic and weakly left-skewed distribution rather than a severe deviation. This is corroborated by the empirical coverage of the cleaned residuals, which lies within a few percent of the nominal Gaussian values for every component (61.9– 70.7% at ±1σ ). The ±1σ bands reported throughout this work are therefore to be understood as an empirically validated dispersion measure. Their actual coverage is close to the nominal Gaussian value, but they are not exact parametric confidence intervals (see Figure 7).

**FIGURE 7:**
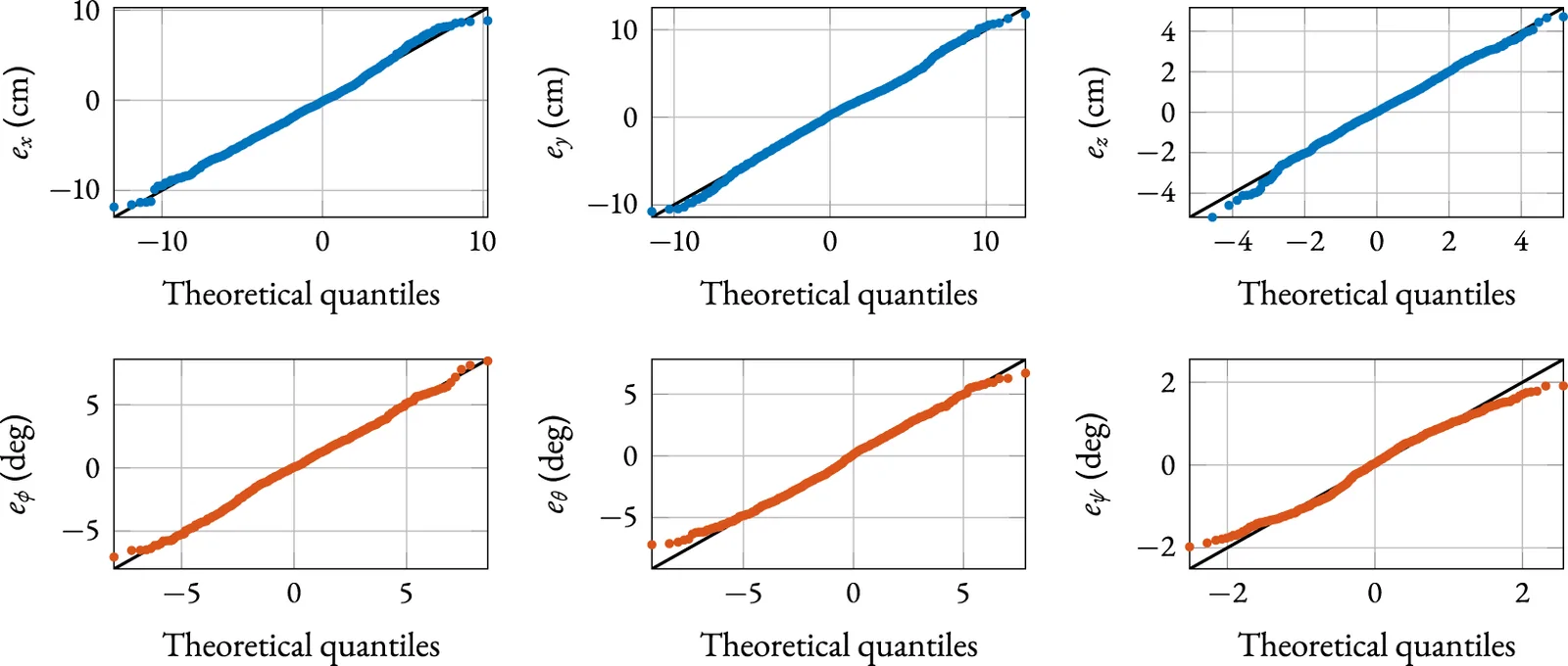
Quantile–quantile plots of the ArUco pose-estimation residuals against a Gaussian reference, computed on the cleaned dataset after Mahalanobis-based outlier rejection (position components, top row; attitude components, bottom row).

Position and attitude errors on the cleaned pool are summarised in Table 2. The 3-D position RMSE was 5.38 cm, with the largest contribution from the horizontal components ( $e_{x}$ : 3.71 cm, $e_{y}$ : 3.60 cm) and a smaller vertical component ( $e_{z}$ : 1.48 cm). Attitude errors remained within 2.49° , 2.61° , and 0.75° RMSE about the camera x , y and z axes, respectively.

**TABLE 2:** ArUco pose-estimation errors relative to motion-capture ground truth, after Mahalanobis-based outlier rejection ( n=1285 frames, N=14 sessions).

Statistic	$e_{x}$ (m)	$e_{y}$ (m)	$e_{z}$ (m)	$e_{\mathrm{xyz}}$ (m)	$e_{\phi }$ (deg)	$e_{\theta }$ (deg)	$e_{\psi }$ (deg)
RMSE	0.037	0.036	0.015	0.054	2.49	2.61	0.75
Mean	− 0.013	0.005	0.003	0.048	0.30	− 0.67	0.02
Std	0.035	0.036	0.014	0.024	2.47	2.53	0.75

The full 6×6 covariance matrix of the error vector, estimated on the cleaned dataset, is given by

$$\Sigma_{\text{pose}}=\left[\begin{array}{cccccc} 11.978 & 1.357 & 0.662 & 0.835 & 4.099 & 0.368\\ 1.357 & 12.690 & -0.326 & 2.607 & 0.408 & 0.338\\ 0.662 & -0.326 & 2.094 & 0.038 & 0.668 & -0.042\\ 0.835 & 2.607 & 0.038 & 6.106 & 0.603 & -0.148\\ 4.099 & 0.408 & 0.668 & 0.603 & 6.378 & 0.029\\ 0.368 & 0.338 & -0.042 & -0.148 & 0.029 & 0.568 \end{array}\right],$$ (14)

where rows and columns are ordered as $\left(e_{x},e_{y},e_{z},e_{\phi },e_{\theta },e_{\psi }\right)$ . The position block (upper-left 3×3 ) is expressed in ${\text{cm}}^{2}$ , the attitude block (lower-right 3×3 ) in ${\text{deg}}^{2}$ , and the position–attitude cross-blocks in cm ⋅ deg.

This characterisation was obtained in a controlled environment, as installing an external ground-truth system in the ALICE cavern is impractical given the access and operational constraints of the experimental area. The marker layout, the blimp platform, the camera and the full pose-estimation pipeline were replicated identically between the two settings, preserving the dominant error drivers of ArUco-based estimation. Ambient illumination and partial occlusion, which do differ, act primarily on the detection stage rather than on the accuracy of accepted detections, and therefore manifest mainly as a reduced number of valid frames. Equation 14 is therefore to be understood as a representative estimate of the *in-situ* uncertainty, which may differ to some extent from the values observed under actual operating conditions.

#### 2.4.2 Uncertainty propagation

To estimate the covariance matrix of the measured magnetic field vector, denoted by $\Sigma_{\text{tot}}$ , the uncertainties associated with both the localisation system and the Hall sensor are propagated through the magnetic-field measurement model. No distributional form is assumed for the individual uncertainty sources: the first-order propagation law operates on variances and requires only that the standard uncertainty of each contribution be defined. Type A contributions (pose) are characterised by their empirical covariance, while Type B contributions (Hall sensor) are modelled as rectangular and converted to standard uncertainties via $u=a/\sqrt{3}$ .

The vector q contains all quantities contributing to the uncertainty quantification, including localisation errors and sensor calibration uncertainties. The uncertain variables are collected in

$$q={\left[\begin{array}{cc} q_{\text{pose}}^{⊤} & q_{\text{hall}}^{⊤} \end{array}\right]}^{⊤}\in R^{9},$$ (15)

with

$$q_{\text{pose}}={\left[\begin{array}{cccccc} x & y & z & \phi & \theta & \psi \end{array}\right]}^{⊤},$$ (16)

representing the ArUco-based estimate of the camera pose in $F_{G}$ , where (ϕ,θ,ψ) are the XYZ Euler angles of RcamG , the same parametrisation in which $\Sigma_{\text{pose}}$ of Equation 14 is estimated, and

qhall=BSxBSyBSz⊤, (17)

representing the raw Hall measurements expressed in the sensor frame, so that qhall≡BSmeas .

The residual ΔB of Equation 9 is a nonlinear function of these n=9 uncertain variables collected in Equation 15:

ΔB=fq=RSALICEqpose qhall−BsimALICEpSqpose, (18)

where $p_{S}\left(q_{\text{pose}}\right)$ and RALICES(qpose) are the sensor position and orientation obtained from the transformation chain of Equation 7. The pose enters f through two distinct mechanisms: the orientation rotates the measured field vector into $F_{\text{ALICE}}$ , while the position determines the point at which the simulated field is sampled.

Linearising f about the nominal operating point $\hat{q}$ yields

$$\Delta B\approx f\left(\hat{q}\right)+J\;\delta q,\;\delta q=q-\hat{q},$$ (19)

where $\delta q\in R^{9}$ collects the deviations of the uncertain variables from their nominal values, modelled as a zero-mean random vector with covariance $\Sigma_{q}$ , and

$$J={\left.\frac{\partial f}{\partial q}\right|}_{\hat{q}}=\left[\begin{array}{cc} {\left.\frac{\partial f}{\partial q_{\text{pose}}}\right|}_{\hat{q}} & {\left.\frac{\partial f}{\partial q_{\text{hall}}}\right|}_{\hat{q}} \end{array}\right]=\left[\begin{array}{cc} J_{\text{pose}} & J_{\text{hall}} \end{array}\right]\in R^{3\times 9},$$ (20)

is the Jacobian matrix of the linearisation in Equation 19, evaluated at the nominal operating point.

An analytical expression for f with respect to the pose variables is not available, since ^ALICE^ $B_{\text{sim}}$ is known only on the discrete CST grid. The corresponding block $J_{\text{pose}}\in R^{3\times 6}$ is therefore evaluated numerically using central finite differences on the full expression of Equation 18:

$${\left[J_{\text{pose}}\right]}_{:,\;j}=\frac{f\left(\hat{q}+\epsilon_{j}\;e_{j}\right)-f\left(\hat{q}-\epsilon_{j}\;e_{j}\right)}{2\;\epsilon_{j}},\;j=1,\ldots ,6,$$ (21)

where $e_{j}\in R^{9}$ is the j -th canonical basis vector, so that for j=1,…,6 only the pose entries of $\hat{q}$ are perturbed while the Hall block is held at its nominal value, and $\epsilon_{j}$ is the finite-difference step associated with the j -th variable (1 mm for the position components, 0.01° for the Euler angles). The two halves of the block have distinct physical meanings: the position columns reduce to −∇BALICEsim , so that their magnitude is governed by the local field gradient, whereas the attitude columns combine the rotation of the measured vector with the displacement of the sensor produced by the camera-to-sensor lever arm.

In contrast, the simulated field does not depend on the Hall measurement, so the residual depends on BSmeas only through the rotation RALICES of Equation 8. The corresponding Jacobian block, $J_{\text{hall}}\in R^{3\times 3}$ , therefore admits a closed-form expression and does not require finite-difference approximation:

Jhall=∂f∂qhallq^=∂ ΔB∂ BSmeasq^pose∂ BSmeas∂ qhall=RALICES I3=RALICES. (22)

The covariance matrix $\Sigma_{q}$ is assembled consistently with the partition of q into the pose and Hall blocks of Equations 16, 17, adopted in Equation 20, combining the empirical pose covariance $\Sigma_{\text{pose}}$ of Equation 14 with the Hall covariance $\Sigma_{\text{hall}}$ of Equation 13. Since localisation errors and sensor noise originate from physically unrelated processes, no correlation is assumed between the two groups of variables, so that

$$\Sigma_{q}=\left[\begin{array}{cc} \Sigma_{\text{pose}} & 0\\ 0 & \Sigma_{\text{hall}} \end{array}\right]\in R^{9\times 9}.$$ (23)

In the assembly of Equation 23, $\Sigma_{\text{pose}}$ is converted to the units in which the Jacobian is evaluated ( ${\text{m}}^{2}$ , ${\text{rad}}^{2}$ and m ⋅ rad), consistent with the finite-difference steps of Equation 21; Equation 14 reports it in cm and degrees for readability.

Using first-order covariance propagation, the covariance matrix of the residual field vector is obtained as

$$\Sigma_{\text{tot}}=J\;\Sigma_{q}\;J^{⊤}.$$ (24)

The resulting 3×3 covariance matrix provides the uncertainty associated with each component of the residual field vector, jointly accounting for pose-localisation uncertainty (including its internal correlations) and Hall-sensor measurement uncertainty propagated through the same rotation that maps the sensor frame into the ALICE frame (Equation 22).

Because $\Sigma_{q}$ is block-diagonal (i.e., tracking errors and sensor noise are statistically independent), the total covariance matrix $\Sigma_{\text{tot}}$ (Equation 24) can be linearly decomposed into two distinct components: one arising strictly from the propagated pose uncertainty $\left(\Sigma_{\text{pose}}\right)$ and one from the sensor’s intrinsic uncertainty $\left(\Sigma_{\text{hall}}\right)$ . Evaluating these contributions allows one to identify whether the dominant source of error at any given time originates from the visual tracking system or the magnetic sensor itself.

#### 2.4.3 Validation metrics

Discrepancies between the measurements and the electromagnetic simulation are quantitatively evaluated using four global validation metrics: the mean residual error $\left(\bar{\Delta B_{i}}\right)$ , the root-mean-square error $\left({RMSE}_{i}\right)$ , the coefficient of determination $\left(R_{i}^{2}\right)$ , and the compliance percentage $\left(P_{i}\right)$ .

First, the mean residual error is computed for each axis to assess any systematic offset or bias:

$$\bar{\Delta B_{i}}=\frac{1}{N}\overset{N}{\underset{k=1}{\sum }}\left(B_{\text{meas},i}^{\;k}-B_{\text{sim},i}^{\;k}\right),\;i=x,y,z,$$ (25)

where N is the number of measurement points. In the static experiment, N=20 , corresponding to the 20 ArUco measurement locations, whereas in the dynamic experiment N is the total number of localised measurement samples acquired during the trajectory.

Second, the root-mean-square error is used to quantify the average magnitude of the deviation:

$${RMSE}_{i}=\sqrt{\frac{1}{N}\overset{N}{\underset{k=1}{\sum }}{\left(B_{\text{meas},i}^{\;k}-B_{\text{sim},i}^{\;k}\right)}^{2}},\;i=x,y,z.$$ (26)

Third, the coefficient of determination is evaluated to assess how well the simulation tracks the variations in the measured data:

$$R_{i}^{2}=1-\frac{\sum_{k=1}^{N}{\left(B_{\text{meas},i}^{\;k}-B_{\text{sim},i}^{\;k}\right)}^{2}}{\sum_{k=1}^{N}{\left(B_{\text{meas},i}^{\;k}-{\bar{B}}_{\text{meas},i}\right)}^{2}},\;i=x,y,z,$$ (27)

where ${\bar{B}}_{\text{meas},i}$ is the mean measured value of the i -th field component over all measurement locations.

Finally, to assess the statistical consistency within the estimated uncertainty, the compliance percentage $P_{i}$ is calculated for each component i=x,y,z :

$$P_{i}=\frac{100}{N}\overset{N}{\underset{k=1}{\sum }}C_{i}^{k},\;i=x,y,z$$ (28)

where $C_{i}^{k}$ is a binary indicator function that verifies whether the simulation falls within the $\pm \sigma_{\text{tot}}$ uncertainty bound around the measured value at the specific point k :

$$C_{i}^{k}=\left\{\begin{array}{cc} 1 & \text{if }\left|B_{\text{sim},i}^{\;k}-B_{\text{meas},i}^{\;k}\right|\leq 1\sigma_{\text{tot},i}^{\;k}\\ 0 & \text{otherwise} \end{array}\right.,\;i=x,y,z$$ (29)

where $\sigma_{\text{tot},i}^{\;k}$ denotes the combined standard uncertainty associated with the i -th component at measurement point k . In the static validation only the Hall-sensor contributions enter $\sigma_{\text{tot}}$ , whereas in the dynamic validation both the Hall-sensor and the pose-localisation contributions are included.

Alongside these metrics, the tables report the mean standard uncertainties ${\bar{\sigma }}_{\text{pose},i}$ , ${\bar{\sigma }}_{\text{hall},i}$ and ${\bar{\sigma }}_{\text{tot},i}$ , obtained by averaging the corresponding per-point quantities over the N measurement points of each dataset,

$${\bar{\sigma }}_{•,i}=\frac{1}{N}\overset{N}{\underset{k=1}{\sum }}\sigma_{•,i}^{\;k},\;•\in \left\{pose,\;hall,\;tot\right\},\;i=x,y,z,$$ (30)

where each $\sigma_{•,i}^{\;k}$ is the square root of the corresponding diagonal entry of the covariance matrix evaluated at point k .

## 3 Results

### 3.1 CST simulation results

The field-line distribution obtained from the CST simulation is shown in Figure 8.

**FIGURE 8:**
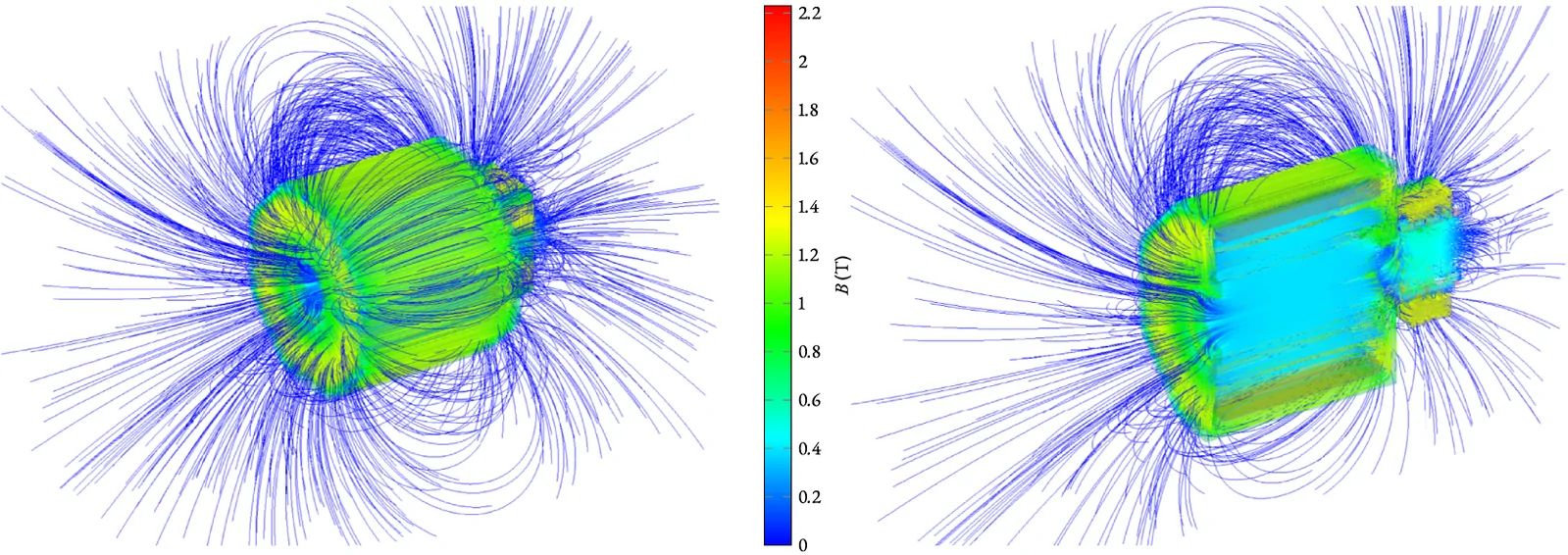
CST Studio Suite simulation of the ALICE detector magnetic field. (Left) Full detector view showing the B-field streamlines. (Right) Cross-section view illustrating the uniformity of field lines inside the L3 solenoid, oriented along the beam-pipe direction ( y -direction), and the fringe field extending into the cavern volume.

To verify the correctness of the electromagnetic model, the contributions of the L3 solenoid and the forward dipole were simulated independently and compared with their nominal magnetic flux densities. Figure 9 shows the resulting field components along the beam axis ( $x_{\text{ALICE}}=0$ , $z_{\text{ALICE}}=0$ ). As expected, the L3 solenoid contributes predominantly to the $B_{y}$ component, while the forward dipole primarily generates the $B_{x}$ component. The simulated field reaches the nominal 0.5 T at the centre of the L3 solenoid, whereas the forward dipole peak field is approximately 3% lower than its nominal value of 0.666 T. This discrepancy was considered sufficiently small for the purposes of the present study.

**FIGURE 9:**
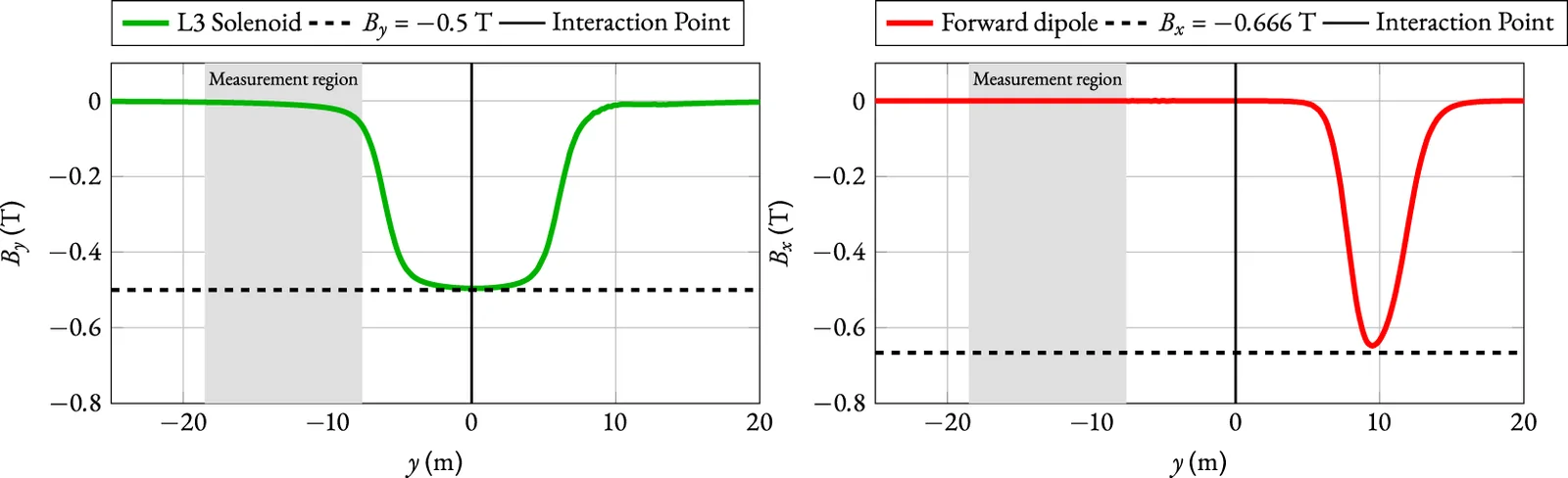
Magnetic field components obtained from the CST simulation along the ALICE beam axis ( $x_{\text{ALICE}}=0$ , $z_{\text{ALICE}}=0$ ). The left panel shows the $B_{y}$ component generated by the L3 solenoid, while the right panel shows the $B_{x}$ component generated by the forward dipole. The dashed horizontal line indicates the nominal magnetic field value of each magnet, and the solid vertical line marks the interaction point $\left(y_{\text{ALICE}}=0\right)$ . The grey shaded region highlights the measurement area where the experimental campaigns were performed.

### 3.2 Static test validation

The results of the static magnetic field measurements described in Section 2.3.4 are shown in Figure 10, which compares the measured and simulated magnetic field components together and shows the corresponding residuals. Error bars represent the Hall-sensor uncertainty $\left(\pm 1\sigma_{\text{hall},i}^{k}\right)$ associated with each of the 20 measurement locations. The corresponding global validation metrics (Section 2.4.3 Equations 25–29) are summarised in Table 3, together with the average Hall-sensor uncertainty (Equation 30).

**FIGURE 10:**
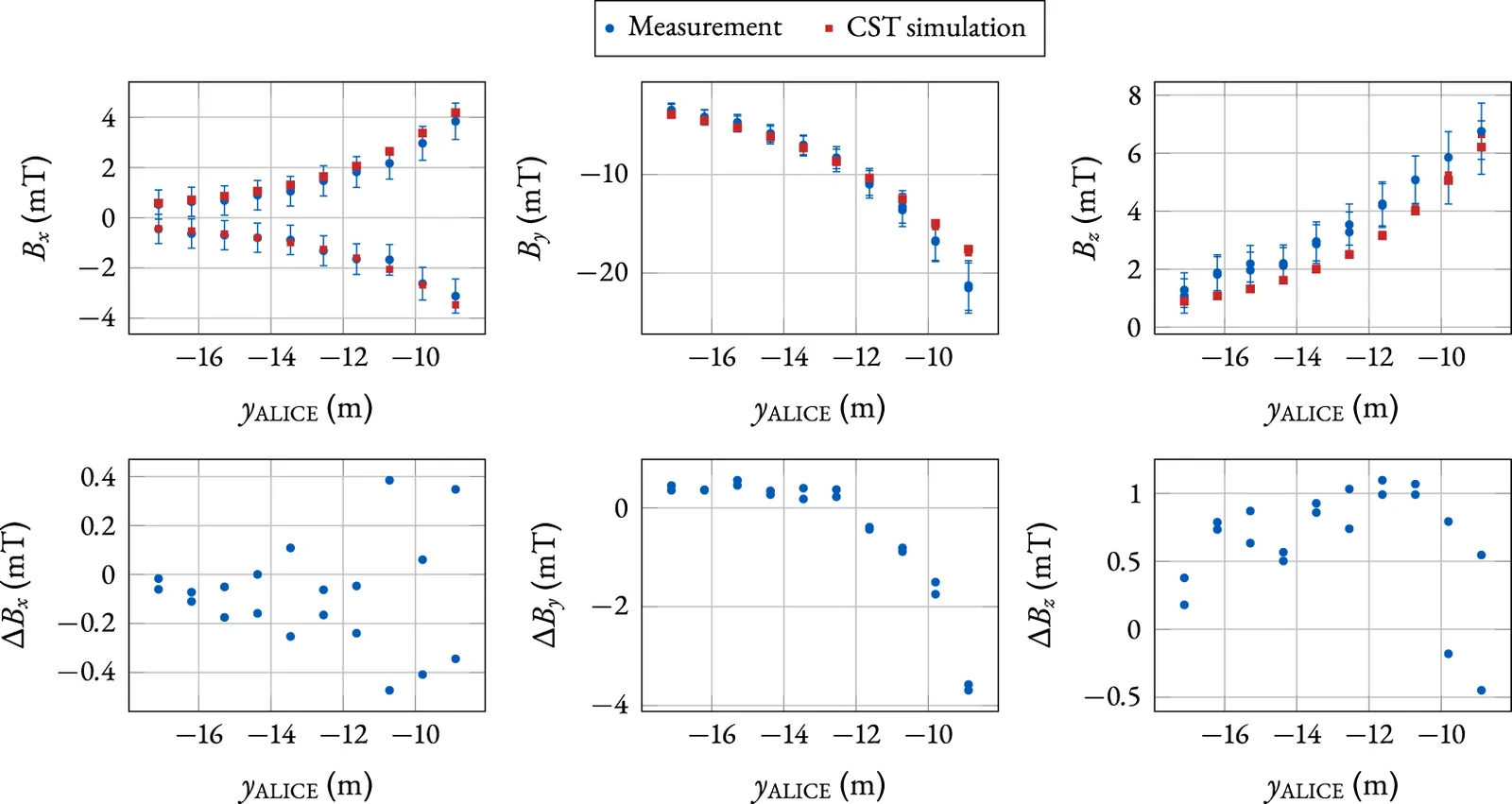
Static test comparison between the measured and CST-simulated magnetic field components $B_{x}$ , $B_{y}$ , and $B_{z}$ along the $y_{\text{ALICE}}$ axis (top row). Error bars indicate the $\pm \sigma_{\text{hall},i}$ uncertainty of the Hall-sensor measurements. The bottom row shows the corresponding residuals $\Delta B_{i}=B_{i}^{\text{meas}}-B_{i}^{\text{CST}}$ for each field component.

**TABLE 3:** Global validation metrics for the residuals between Hall-sensor measurements and CST simulations at the 20 ArUco locations during the static test.

Component	${\bar{\sigma }}_{\text{hall},i}$ (mT)	${RMSE}_{i}$ (mT)	$\bar{\Delta B_{i}}$ (mT)	$R_{i}^{2}$	$P_{i}$ (%)
$B_{x}$	0.611	0.227	− 0.087	0.9836	100
$B_{y}$	1.287	1.326	− 0.436	0.9446	90
$B_{z}$	0.721	0.767	0.654	0.7919	40

Each static value is the arithmetic mean of the N samples acquired over the corresponding stationary window (median N=4139 , median duration 30.9 s), after discarding an initial transient. Averaging suppresses only the random contribution, so the noise term $\sigma_{\text{noise},i}$ entering Equation 12, taken from the manufacturer specification throughout this work, is evaluated for the mean as $\sigma_{\text{noise},i}/\sqrt{N}$ , yielding 1.9 µT on the x and y axes and 1.1 µT on z . The sensitivity and offset terms, being systematic within the window, apply to the mean unchanged. The budget is therefore entirely governed by the systematic contributions: across the twenty static points, the noise accounts for less than 0.01% of the total variance, while the offset term of Equation 11 dominates at low field magnitudes (up to 99.2%) and the sensitivity term of Equation 10 at the largest ones.

The independence of consecutive samples, required for the $1/\sqrt{N}$ reduction, was verified on all twenty acquisitions: the lag-one autocorrelation of the residuals about the window mean remains below 0.05 in magnitude for every field component, and identical consecutive readings account for less than 0.3% of the samples. Consistently, the measured within-window standard deviation (107 µT on the x and y axes, 57 µT on z ) is close to, and slightly below, the RMS noise specified by the manufacturer for the same configuration. This measured value is reported solely as an independent consistency check on the datasheet figure and does not enter the propagation, the manufacturer specification being retained throughout as the more conservative choice.

Among the three field components, $B_{x}$ exhibits the strongest agreement with the simulation, achieving an ${RMSE}_{x}$ of 0.23 mT and a coefficient of determination of $R_{x}^{2}=0.984$ . The largest discrepancy is observed for $B_{y}$ , whose ${RMSE}_{y}$ reaches 1.33 mT despite maintaining a high coefficient of determination $\left(R_{y}^{2}=0.945\right)$ . The $B_{z}$ component exhibits an intermediate ${RMSE}_{z}$ of 0.77 mT but the lowest coefficient of determination $\left(R_{z}^{2}=0.792\right)$ , indicating a larger spread of the residuals. Small systematic offsets are observed for all three components, the largest occurring in $B_{z}$ , where the mean residual $\bar{\Delta B_{z}}=+0.65$ mT indicates that the measured field is consistently higher than the corresponding CST prediction.

The compliance percentage $P_{i}$ further confirms the excellent agreement for the $B_{x}$ and $B_{y}$ components. The simulated field lies within the Hall-sensor uncertainty bounds for all 20 measurement locations in $B_{x}$ (100%) and for 18 out of 20 locations in $B_{y}$ (90%). In contrast, only 8 out of 20 locations (40%) satisfy the same criterion for $B_{z}$ . These results indicate that the discrepancy in the simulation model is considerably more pronounced for the z -component than for the other two. This is consistent with a modelling error potentially associated with the ferromagnetic yoke in the CST electromagnetic model, although the static campaign cannot fully exclude residual contributions from sensor offset, sensor alignment, or coordinate-transformation errors, which were not independently characterised in this analysis.

### 3.3 Dynamic flight test validation

The 3D trajectories recorded during the flight campaign are shown in Figure 11, colour-coded according to the measured magnetic field magnitude, |BALICEmeas| . The trajectories were confined to the same region instrumented with ArUco markers used during the static measurement campaign, ensuring continuous pose estimation.

**FIGURE 11:**
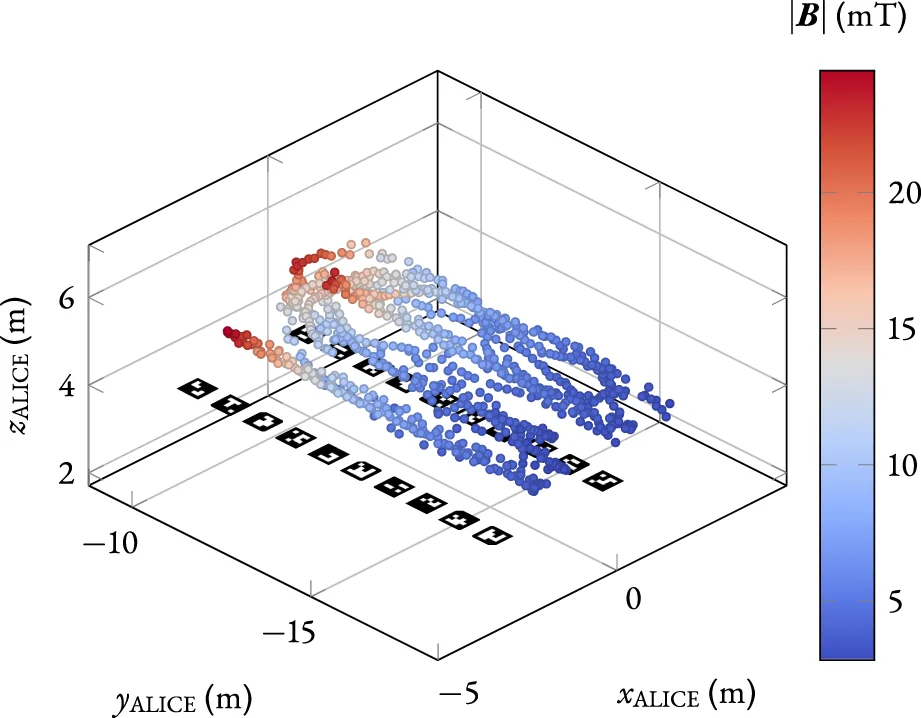
3D flight trajectories of the blimp in the ALICE cavern, colour-coded by total magnetic field magnitude |B| (mT). ArUco markers are shown at 3× their actual size for visibility.

As already observed in the static test, a clear spatial gradient is visible along the $y_{\text{ALICE}}$ axis (beam direction): |BALICEmeas| increases as the blimp approaches $y_{\text{ALICE}}\approx -8$ m near the L3 solenoid doors, and decreases toward $y_{\text{ALICE}}\approx -18$ m further into the open cavern.

The measured and simulated profiles as a function of $y_{\text{ALICE}}$ , together with the per-component residuals $\Delta B_{i}$ , are shown in Figure 12 for all four flights.

**FIGURE 12:**
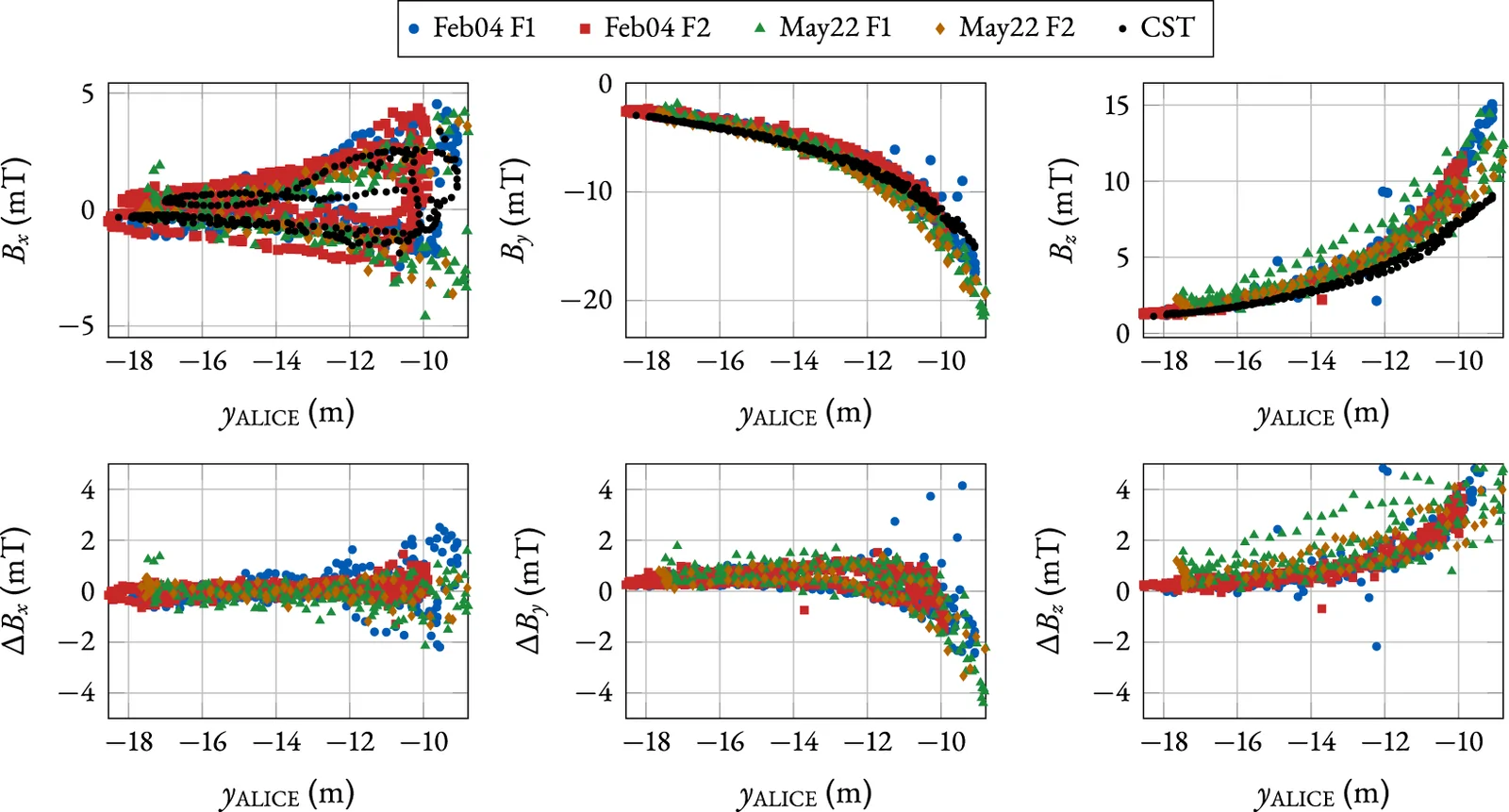
Dynamic flight test comparison between Hall-sensor measurements and CST simulations along the $y_{\text{ALICE}}$ axis for the three magnetic field components ( $B_{x}$ , $B_{y}$ , $B_{z}$ ). The upper row shows the field profiles for the four flights, while the lower row reports the corresponding residuals $\Delta B_{i}=B_{i}^{\text{meas}}-B_{i}^{\text{CST}}$ for each field component.

Table 4 reports the validation metrics presented in Section 2.4.3 for each flight, together with the total combined field-measurement uncertainty, ${\bar{\sigma }}_{\text{tot},i}$ , which accounts for both the pose-induced $\left({\bar{\sigma }}_{\text{pose},i}\right)$ and Hall-sensor $\left({\bar{\sigma }}_{\text{hall},i}\right)$ uncertainties. These components are obtained from the diagonal elements of the pose and Hall contributions to $\Sigma_{\text{tot}}$ following the error-propagation procedure described in Section 2.4.2.

**TABLE 4:** Global validation metrics for the residuals between Hall-sensor measurements and CST simulations for each flight during the dynamic flight tests.

Flight	Component	${\bar{\sigma }}_{\text{pose},i}$ (mT)	${\bar{\sigma }}_{\text{hall},i}$ (mT)	${\bar{\sigma }}_{\text{tot},i}$ (mT)	${RMSE}_{i}$ (mT)	$\bar{\Delta B_{i}}$ (mT)	$R_{i}^{2}$	$P_{i}$ (%)
Feb 4 – F1	$B_{x}$	0.245	0.708	0.759	0.716	0.201	0.7655	82.5
	$B_{y}$	0.222	0.958	0.987	0.915	0.214	0.9446	78.9
	$B_{z}$	0.303	0.882	0.934	1.946	1.359	0.7156	53.7
Feb 4 – F2	$B_{x}$	0.226	0.712	0.753	0.408	0.209	0.9192	94.0
	$B_{y}$	0.203	0.936	0.961	0.575	0.302	0.9707	85.6
	$B_{z}$	0.297	0.835	0.889	1.485	1.154	0.7384	52.0
May 22 – F1	$B_{x}$	0.225	0.628	0.676	0.496	0.086	0.9197	88.9
	$B_{y}$	0.184	1.184	1.199	1.708	− 0.428	0.8912	69.6
	$B_{z}$	0.384	0.821	0.913	1.125	0.886	0.8043	59.6
May 22 – F2	$B_{x}$	0.198	0.633	0.672	0.963	0.204	0.6826	90.5
	$B_{y}$	0.160	1.125	1.137	1.463	− 0.295	0.9146	71.6
	$B_{z}$	0.355	0.770	0.855	0.945	0.729	0.8431	67.6
All flights	$B_{x}$	0.229	0.687	0.732	0.589	0.182	0.8563	89.4
	$B_{y}$	0.201	1.008	1.031	1.071	0.080	0.9337	79.3
	$B_{z}$	0.321	0.840	0.904	1.536	1.123	0.7485	55.4

Across all valid points the simulation reproduces the measured field closely for the transverse components, with combined RMSE values of 0.59 mT and 1.07 mT and correspondingly high coefficients of determination ( $R_{x}^{2}=0.856$ , $R_{y}^{2}=0.934$ ). The vertical component $B_{z}$ exhibits both the weakest correlation ( $R_{z}^{2}=0.749$ ) and the largest residuals ( ${RMSE}_{z}=1.54$ mT), together with a consistent positive bias ( $\bar{\Delta B_{z}}\approx +1.12$ mT across all flights).

The pose-induced term ${\bar{\sigma }}_{\text{pose},i}$ is smaller than the Hall-sensor term ${\bar{\sigma }}_{\text{hall},i}$ in every flight and for every component, meaning that the Hall-sensor uncertainty is the dominant contribution to the total uncertainty ${\bar{\sigma }}_{\text{tot}}$ , consistent with the proportional component of the TMAG5273 uncertainty model ( $\propto 0.1155\;|B_{i}|$ , Equation 10), which inflates ${\bar{\sigma }}_{\text{hall}}$ wherever the locally measured field is larger, as is the case for $B_{y}$ inside the cavern.

As in the static case, $B_{x}$ shows the best agreement between measurement and simulation, with the smallest pooled residual ( ${RMSE}_{x}=0.59$ mT) and a bias that is small and consistent across all four flights ( +0.09 to +0.21 mT). Its RMSE remains below ${\bar{\sigma }}_{\text{tot}}$ in three flights out of four, exceeding it only in May 22 – F2 (ratio 1.43). $B_{y}$ shows a bias that changes sign between campaigns ( +0.21 and +0.30 mT in February, −0.43 and −0.30 mT in May) and an RMSE comparable to ${\bar{\sigma }}_{\text{tot}}$ , with a ratio ranging from 0.60 to 1.42 and equal to 1.04 when pooled over all flights. $B_{z}$ remains the clear outlier: the RMSE exceeds ${\bar{\sigma }}_{\text{tot}}$ by a factor of 1.1–2.1 in every flight (1.70 when pooled), and the bias is large, positive and of the same sign in every session ( +0.73 to +1.36 mT), confirming a systematic offset already identified in the static test (Table 3).

This picture is confirmed by the coverage analysis quantified by the compliance percentage $P_{i}$ defined in Section 2.4.3. Pooling all 858 valid points across the four flights, $B_{x}$ satisfies the ± ${\bar{\sigma }}_{\text{tot}}$ bound 89.4% of the time and $B_{y}$ 79.3% of the time, both in line with the static-test coverage of 100% and 90%, respectively. $B_{z}$ coverage is markedly lower, dropping to 55.4% when pooled over all flights, a value comparable to the 40% obtained in the static test. This analysis confirms the outcome of the static test in Section 3.2, with $B_{z}$ exhibiting the largest discrepancy.

It should be noted that, since the samples were collected along continuous flight trajectories, consecutive measurements are spatially and temporally correlated and therefore do not constitute independent observations. The reported sample count (858 points, pooled across the four flights) should thus not be interpreted as an equivalent number of independent validation points. For this reason, the agreement between measured and simulated fields is assessed jointly through the field profiles, bias, RMSE , and compliance percentage $P_{i}$ , rather than relying primarily on the coefficient of determination $R^{2}$ , which is more sensitive to sample correlation.

## 4 Discussion

This work demonstrates the feasibility of deploying a lightweight robotic airship for *in situ* stray magnetic field mapping in a large underground physics facility. Two complementary experimental campaigns were conducted at the ALICE cavern, with the L3 solenoid and forward dipole at nominal current: static measurements at known locations, which isolated the Hall-sensor contribution from the localisation uncertainty, and dynamic flight tests over a surveyed volume of approximately 6×11×3 ${\text{m}}^{3}$ on the A-side of the ALICE cavern, employing the complete measurement pipeline including the ArUco-based pose estimation.

Both campaigns showed strong agreement between measurement and simulation for the transverse components ( $R_{x}^{2}=0.984$ , $R_{y}^{2}=0.945$ statically; $R_{x}^{2}=0.856$ , $R_{y}^{2}=0.934$ dynamically), while $B_{z}$ consistently stood out as the weakest component ( $R_{z}^{2}=0.792$ statically, 0.749 dynamically), with a systematic positive bias. Critically, since this bias appears already in the static campaign, where the ArUco-based pose-estimation contribution is absent, it suggests that the $B_{z}$ discrepancy originates primarily in the electromagnetic model and not in the localisation pipeline. The terms excluded from the propagation of Section 2.4.2, namely, the camera-to-sensor and global-to-ALICE transformations, taken as nominal values from CAD data, and the residual time synchronisation, are fixed for the entire campaign and lack a documented tolerance, so no standard uncertainty can be assigned to them; they are therefore treated as ideal. Unlike the Hall-sensor terms, whose bounds are specified by the manufacturer and are accordingly evaluated as Type B contributions (Section 2.4.1.1), these transformations would act as a bias common to all measurement points rather than as a point-to-point dispersion, and are therefore not captured by the $\pm \sigma_{\text{tot}}$ bands, which quantify dispersion. Given the tolerances of the mechanical assembly and the centimetre-level accuracy of the CAD survey, their contribution is expected to remain limited with respect to the observed bias and is unlikely to account for it on its own. A dedicated sensitivity study, addressing both these transformation terms and the response of the CST model to its geometry and material parameters, would be needed to fully resolve the residual discrepancy and is left as future work.

This discrepancy is most plausibly attributed to limitations in the CST geometry and material selection. Both yokes were modelled using the isotropic B – H curve of AISI 1050 annealed carbon steel from the Granta Selector database, applied uniformly to the L3 return yoke and the forward dipole yoke; the actual yoke materials are not documented, introducing a systematic uncertainty in the fringe-field components most sensitive to the permeability value. In addition, the CST geometry does not include the concrete shielding blocks located adjacent to the solenoid access doors. Since these blocks contain ferromagnetic material, they may locally perturb the magnetic field and contribute to the observed discrepancies. Both effects are expected to alter the fringe field distribution in the A-side region. A parametric sensitivity study, varying the yoke permeability and shielding geometry within the CST model, is left as future work to further quantify and reduce this discrepancy.

On the measurement side, a significant limitation of the current system is the dependence on ArUco marker visibility for absolute localisation: during flight segments in which no marker is in the camera field of view, the sensor position is unknown and the corresponding Hall-sensor readings cannot be spatially georeferenced. This constraint also shaped the flown trajectories, which sampled a relatively narrow corridor in front of the A-side detector shielding blocks rather than the full cavern volume. The four manually piloted flights reported here therefore demonstrate the measurement and localisation methodology within an instrumented test region, and do not constitute a complete survey of the cavern. Extending the marker layout for wider coverage, together with onboard dead-reckoning methods such as visual odometry, IMU integration, and time-of-flight altitude sensing, would help bridge localisation gaps and maximise the fraction of the Hall-sensor time series that can be spatially associated, progressively widening the mapped volume toward the full cavern.

Finally, the current flights were conducted under manual joystick control, which limits the reproducibility and spatial coverage of the trajectories and introduces a dependency on operating during the narrow time windows of LHC technical stops. The most significant planned evolution of the system is therefore the transition to autonomous operation, integrating the ArUco-based localisation pipeline with a closed-loop position controller and a field-aware path planner. This would allow the blimp to autonomously execute pre-programmed survey patterns over progressively wider portions of the cavern, repeat missions with consistency, and function as a continuous monitoring system capable of detecting field anomalies during beam operations without requiring human presence in the cavern. Together, the measurement methodology, localisation pipeline, and uncertainty framework developed in this work constitute the foundation for such a fully autonomous airship-based survey system in underground physics environments.

## Data Availability

The raw data supporting the conclusions of this article will be made available by the authors, without undue reservation.
